# ITGAV and SMAD4 influence the progression and clinical outcome of pancreatic ductal adenocarcinoma

**DOI:** 10.1002/1878-0261.70080

**Published:** 2025-07-30

**Authors:** Daniel K. C. Lee, Keyue Chen, Ryan Loke, Xiang Li, David Liubart, Golam T. Saffi, Jonathan T. S. Chow, Ché M. P. Melo, Lydia To, Leonardo Salmena

**Affiliations:** ^1^ Department of Pharmacology & Toxicology University of Toronto Canada; ^2^ Princess Margaret Cancer Centre University Health Network Toronto Canada

**Keywords:** invasion, ITGAV, PDAC, SMAD4

## Abstract

Pancreatic ductal adenocarcinoma (PDAC) is a very aggressive and lethal malignancy with limited treatment options, a fact that underscores the urgent need for more effective therapies to improve patient outcomes. Preclinical studies have shown promise for αV integrin‐targeted therapies; however, clinical trials have been disappointing, highlighting the need for further research. In this study, we demonstrate that integrin subunit alpha V (ITGAV) signals through both mothers against decapentaplegic homolog 4 (SMAD4)‐dependent or SMAD4‐independent pathways, depending on the genetic context. In *SMAD4*‐positive PDAC cells, ITGAV contributes to the transforming growth factor‐beta (TGF‐β) signaling pathway to regulate proliferation, migration, and invasion. Conversely, in *SMAD4*‐negative PDAC cells, ITGAV influences only proliferation and migration via activation of the mitogen‐activated protein kinase (MAPK)/extracellular signal‐related kinase (ERK) pathway. High levels of *ITGAV* are also associated with poor prognostic outcomes in *SMAD4* wild‐type patients but are not prognostic in *SMAD4* mutant patients. Thus, ITGAV contributes to different patterns of PDAC progression. These findings suggest that stratifying PDAC patients based on both *SMAD4* status and *ITGAV* expression could inform more effective integrin‐targeted treatment strategies.

## Graphical abstract

In SMAD4‐positive pancreatic ductal adenocarcinoma (PDAC), integrin subunit alpha V (ITGAV) activates latent TGF‐β, which binds to the TGF‐β receptor and phosphorylates SMAD2/3. The activated SMAD2/3 forms a complex with SMAD4, and together they translocate to the nucleus, modulating gene expression to promote proliferation, migration, and invasion. In SMAD4‐negative PDAC, ITGAV activates ERK signaling to promote proliferation and migration.

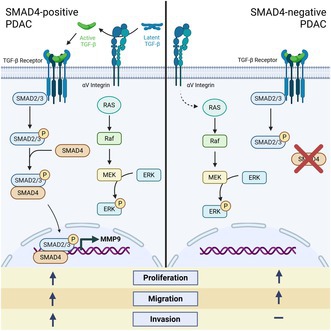

**Abbreviations**
Cas9: CRISPR‐associated protein 9
CRISPR: Clustered Regularly Interspaced Short Palindromic Repeats
ECM: extracellular matrix
ERK: extracellular signal‐regulated kinase
GSEA: gene set enrichment analysis
ITGAV: integrin subunit alpha V
KO: knockout
MAPK: mitogen‐activated protein kinase
MMP: matrix metalloproteinase
PDAC: pancreatic ductal adenocarcinoma
sgRNA: single guide RNA
SMAD4: mothers against decapentaplegic homolog 4
TGF‐β: transforming growth factor‐beta
TβRI: TGF‐β receptor I

## 1 Introduction

Pancreatic ductal adenocarcinoma (PDAC) is among the most aggressive and lethal cancer types, with a five‐year survival rate of less than 10% [1]. It is currently the third‐leading cause of cancer‐related death worldwide and is projected to become the second‐leading cause of cancer death by 2030 [1, 2]. The high mortality of PDAC stems from a lack of early biomarkers, early and aggressive invasion, formation of distant metastases, and poor response to conventional chemotherapy [3]. Surgical resection remains the only potentially curative option; however, only 15–20% of patients are diagnosed with localized disease amenable to surgery at the time of presentation [4]. The majority of PDAC patients receive standard chemotherapy regimens, which include FOLFIRINOX or gemcitabine‐based therapy [4]. Despite these efforts, the significant challenges of chemoresistance and adverse effects restrict the efficacy of treatment [5, 6, 7]. Thus, there remains an urgent need to develop more effective, targeted, and personalized therapies to improve PDAC disease outcome.

Previous work by our group has identified *integrin subunit alpha V* (*ITGAV*) as an essential gene in PDAC cells [8], highlighting a pivotal role for integrin signaling in PDAC progression. Integrins are transmembrane proteins that mediate cell–cell and cell–extracellular matrix (ECM) interactions, crucial for various cellular processes including adhesion, migration, proliferation, and survival [9]. ITGAV, the αV subunit of the integrin family, forms heterodimeric complexes primarily with β1, β3, β5, β6, or β8 subunits, allowing cells to sense and respond to their microenvironmental cues [9]. These interactions enable integrins to transmit signals bidirectionally across the plasma membrane, influencing intracellular signaling cascades that regulate gene expression, cytoskeletal organization, cell migration, and invasion [9]. In cancer, dysregulated integrin signaling, often driven by aberrant expression or activation of specific integrin subunits such as ITGAV, contributes to tumor progression, metastasis, and treatment resistance [10, 11, 12, 13, 14, 15]. However, despite their critical roles in cancer biology and preclinical insights, targeting integrins in the clinic has generally not translated into effective treatments [16, 17, 18, 19, 20, 21].

In this study, we investigated the role of ITGAV across different genetic subtypes of PDAC to identify putative subtype‐specific therapeutic strategies. Our findings show that ITGAV activates signal transduction via the transforming growth factor‐beta (TGF‐β) or mitogen‐activated protein kinase (MAPK)/extracellular signal‐regulated kinase (ERK) signaling pathway, with the outcome influenced by the status of *mothers against decapentaplegic homolog 4 (SMAD4)*, leading to varying impacts on cell invasion. Our findings suggest that stratifying PDAC patients based on *SMAD4* status could underpin more targeted and effective treatment strategies with integrin‐targeted agents. Our findings suggest that stratifying PDAC patients based on *SMAD4* status hold promise of more targeted and effective treatment strategies with integrin‐targeted agents.

## 2 Materials and methods

### 2.1 Cell line maintenance

BxPC‐3 (RRID: CVCL_0186), HEK293T (RRID: CVCL_0063), HPAC (RRID: CVCL_3517), PANC‐1 (RRID: CVCL_0480), and PK‐1 (RRID: CVCL_4717) cells were obtained from ATCC and maintained in DMEM (Wisent Inc., 319‐005‐CL) supplemented with 10% fetal bovine serum (FBS) (Wisent Inc., 080‐150, Saint‐Jean‐Baptiste, QC, Canada) and 1% penicillin/streptomycin (Wisent Inc., 450‐201‐EL). Cells were dissociated using 0.25% Trypsin–EDTA (Wisent Inc., 325‐043‐EL). All cell lines were maintained at 37°C and 5% CO_2_. Cell lines were routinely verified to be mycoplasma‐free using the PlasmoTest Mycoplasma Detection Kit (InvivoGen, San Diego, CA, USA) and authenticated by STR analysis (The Centre for Applied Genomics, SickKids, Toronto, Ontario, Canada) within three years of use for this study.

### 2.2 Individual sgRNA cloning

sgRNAs targeting *LacZ* or *ITGAV* were designed using CRISPick [22]. sgRNA target sequences include LacZ: CCCGAATCTCTATCGTGCGG; ITGAV‐1: AGTTCTCCAATGGTACAATG; ITGAV‐2: GCCTTAACAATCAATGTCAG. sgRNAs were cloned into either lentiGuide‐Puro (Addgene Plasmid #52963) or lentiCRISPRv2 (Addgene Plasmid #52961) as previously described [8].

### 2.3 Generation of ITGAV overexpression constructs

To generate *ITGAV* overexpression constructs, the *ITGAV* open reading frame sequence (NM_002210.5; 3162 bp) was synthesized and cloned into pcDNA3.1(+)‐C‐6XHis (GenScript, Piscataway, NJ, USA). To generate sgRNA‐resistant *ITGAV*, site‐directed mutagenesis was performed on this construct using the Q5 Site‐Directed Mutagenesis Kit (NEB, E0554). The first round of mutagenesis targeted the sgRNA‐ITGAV #1 site using pcDNA3.1(+)‐C‐6XHis and specific mutagenic primers (forward—cattggcgaactGAGATGAAACAGGAGC; reverse—atacagcggcgCACAGGCCAAAATTTTATC). The resulting construct served as the template for a second round of mutagenesis of sgRNA‐ITGAV #2 with a new set of primers (forward—gattgtaaaggcTCAGAATCAAGGAGAAGG; reverse—agggtcagcggGTTGTCATCCCCAATATAG). All mutagenic sequences were based on codon‐optimized *ITGAV* and verified to have no predicted on‐target activity using the Rule Set 2/Azimuth 2 algorithm [23, 24]. Wild‐type and mutant ITGAV‐C‐6His coding sequences were subcloned to replace Cas9 in the lenti‐Cas9‐Blast construct (Addgene Plasmid #52962) to generate lenti‐ITGAV‐C‐6XHis‐Blast constructs.

### 2.4 Lentivirus production

Lentivirus packaged with Cas9, SMAD4, or sgRNA targeting *LacZ* or *ITGAV* was produced as previously described [8].

### 2.5 Cas9 cell line generation

Clonal HPAC and PANC‐1 Cas9 cells were generated by limiting dilution as previously described [8].

### 2.6 *ITGAV* knockout (KO) cell line generation

3 × 10^5^ HPAC or PANC‐1 Cas9 cells were infected with a virus expressing either lentiGuide‐LacZ‐puro or lentiGuide‐ITGAV‐puro, as described above. Similarly, 3 × 10^5^ BxPC‐3 or PK‐1 cells were infected with a virus expressing either lentiCRISPRv2‐LacZ‐puro or lentiCRISPRv2‐ITGAV‐puro. After a 24‐h recovery period, infected cells were selected with 2 μg·mL^−1^ puromycin for 48 h and then plated in various molecular or phenotypic assays.

### 2.7 BxPC‐3 SMAD4 cell line generation

3 × 10^5^ BxPC‐3 cells were infected with pLVX‐IRES Hyg SMAD4 (Addgene #107128) virus, as described above. After a 24‐h recovery period, infected cells were selected with 1500 μg·mL^−1^ hygromycin (Gibco, 10 687 010, Waltham, MA, USA) for 7–10 days. Once stable cell lines were generated, SMAD4 expression was verified by western blot, and the cells were subsequently plated in various molecular or phenotypic assays.

### 2.8 Growth curves/proliferation assay

For all proliferation experiments, 5 × 10^3^ cells were seeded per well in a 12‐well plate. For GLPG0187 experiments, cells were allowed to attach overnight and then treated with either vehicle or GLPG0187 (MedChemExpress, HY‐100506) (prepared in DMSO and diluted in DMEM). Cells were fixed with 10% formalin (Sigma‐Aldrich, HT501128) and stained with 0.1% crystal violet in 20% methanol at the indicated timepoints. Each well was washed with Milli‐Q water and left overnight to dry at room temperature. After imaging of wells, crystal violet was solubilized with 1 mL of 10% acetic acid, and the absorbance at 595 nm was measured using a SpectraMax M3 spectrophotometer. Relative cell growth was calculated by normalizing to day 0. Statistical analysis of three independent experiments (mean ± SEM) was assessed using a two‐way ANOVA with Dunnett's *post hoc* in graphpad prism 9.3.0.

### 2.9 Transwell migration assay

For *ITGAV* KO experiments, 2 × 10^5^ HPAC Cas9, PANC‐1 Cas9, BxPC‐3, or PK‐1 cells were seeded in the upper chambers of 12‐well ThinCerts with 8‐μm pores (Greiner Bio‐One, 665 638, Monroe, NC, USA) in DMEM without FBS. In the case of TGF‐β1 experiments, cells were allowed to attach (~ 4 h) and then either vehicle or TGF‐β1 (Sigma‐Aldrich, T7039, St. Louis, MO, USA ) (prepared in water and diluted in DMEM without FBS) was added. For GLPG0187 experiments, 2 × 10^5^ HPAC, PANC‐1, BxPC‐3, or PK‐1 cells were seeded in the upper chamber of 12‐well ThinCerts with 8‐μm pores in DMEM. Cells were allowed to attach (~ 4 h) and then either vehicle or GLPG0187 (diluted in DMEM without FBS) was added. Cells were allowed to migrate for 48 h toward DMEM with 10% FBS and 1% penicillin/streptomycin in all experiments.

For all experiments, nonmigrated cells were removed from the upper chamber with cotton swabs. Cells adhering to the bottom side of the chamber were fixed with 10% formalin and stained with 0.1% crystal violet in 20% methanol. Each membrane was washed with Milli‐Q water and left overnight to dry at room temperature. The bound crystal violet solution was solubilized with 1 mL of 10% acetic acid for 30 min and the absorbance at 595 nm was measured using a SpectraMax M3 Spectrophotometer. Relative cell migration was calculated by correcting for background absorbance and normalizing to sgRNA‐*LacZ* or vehicle control. Statistical analysis of three independent experiments (mean ± SEM) was assessed using a one‐way ANOVA with Dunnett's or Sidak's post hoc in graphpad prism 9.3.0.

### 2.10 Transwell invasion assay

The upper chamber of 24‐well ThinCerts with 8‐μm pores (Greiner Bio‐One, 662 638) was coated with 100 μL of cold Matrigel diluted in DMEM without FBS and then placed in the 37 °C incubator for polymerization. For *ITGAV* KO experiments, 1 × 10^5^ HPAC Cas9, PANC‐1 Cas9, BxPC‐3, BxPC‐3 SMAD4, or PK‐1 cells were seeded in the upper chambers in DMEM without FBS. In the case of TGF‐β1 experiments, cells were allowed to attach (~ 4 h) and then either vehicle or TGF‐β1 (diluted in DMEM without FBS) was added. For GLPG0187 experiments, 1 × 10^5^ HPAC, PANC‐1, BxPC‐3, or PK‐1 cells were seeded in the upper chambers. Cells were allowed to attach (~ 4 h) and then either vehicle or GLPG0187 (diluted in DMEM without FBS) was added. For all experiments, cells were allowed to invade for 48 h toward DMEM with 10% FBS and 1% penicillin/streptomycin. Noninvaded and invaded cells were processed as described above. Relative cell invasion was calculated by correcting for background absorbance and normalizing to sgRNA‐*LacZ* or vehicle control. Statistical analysis of three independent experiments (mean ± SEM) was assessed using a one‐way ANOVA with Dunnett's or Sidak's *post hoc* in graphpad prism 9.3.0.

### 2.11 Western blotting

Western blots were conducted as previously described [8]. Antibodies used in this study include the following: β‐actin (1 : 10000; CST #4967), Cas9 (1 : 1000; CST #14697), ITGAV (1 : 50000; Abcam ab179475), p44/42 MAPK (1 : 2000; CST #4695), phospho‐p44/42 MAPK (Thr202/Tyr204) (1 : 2000; CST #9101), phospho‐SMAD3 (1 : 1000; CST #9520), SMAD3 (1 : 1000; CST #9523), and SMAD4 (1 : 2000; CST #46535). Analysis was done using relative quantitation, with all samples normalized to β‐actin expression. Statistical analysis of three independent experiments (mean ± SEM) was assessed using a one‐way ANOVA with Dunnett's or Sidak's *post hoc* in graphpad prism 9.3.0.

### 2.12 Quantitative polymerase chain reaction (qPCR)

RNA was extracted from cells using the QIAGEN RNeasy Mini Kit (Qiagen, 74 104, Germantown, MD, USA), according to the manufacturer's protocol. Genomic DNA elimination and cDNA synthesis were performed using the SuperScript IV VILO Master Mix (Invitrogen, 11 766 050, Waltham, MA, USA). qPCR was performed using TaqMan Fast Advanced Master Mix (Applied Biosystems, 4 444 965, Waltham, MA, USA) on the QuantStudio 3 Real‐Time PCR System. TaqMan assays used in this study include: *Actb* (Hs03023943_g1) and *Mmp9* (Hs00957562_m1). Analysis was done using the delta–delta *C*_*t*_ (ΔΔ^*C*^_*t*_) method, with all samples normalized to *Actb* expression. Statistical analysis of three independent experiments (mean ± SEM) was assessed using a one‐way ANOVA with Dunnett's or Sidak's *post hoc* in graphpad prism 9.3.0.

### 2.13 Datasets

TCGA‐PAAD RNA‐seq and corresponding clinical data were downloaded from the Genomic Data Commons (GDC) data portal (https://portal.gdc.cancer.gov/) on May 11th, 2021. RNA‐seq data were log_2_ transformed and normalized using an in‐house Python script. Normalized microarray data from the Badea (GSE15471), Moffit (GSE71729), Pei (GSE16515), Sandhu (GSE60980), Yang (GSE62452), and Zhang (GSE28735) datasets were downloaded from the Gene Expression Omnibus (GEO) database (https://www.ncbi.nlm.nih.gov/geo/) on May 17th, 2021. CPTAC‐PDAC proteomic data were downloaded from the Proteomic Data Commons (PDC) data portal (https://pdc.cancer.gov/pdc/) on August 9th, 2023. Peptide intensity was median normalized using an in‐house Python script.

### 2.14 Survival analysis

High and low *ITGAV* expression levels were defined using the Subgroup Identifier (SubID) method, which identified the top 30% of samples as having significantly elevated expression compared to the remainder of the cohort [25]. The gene expression association to survival was plotted using the Kaplan–Meier method and analyzed using the log‐rank test. Survival analysis with gene expression data from the TCGA‐PAAD patient dataset was plotted in graphpad prism 9.3.0.

### 2.15 Gene expression analysis

Normalized *ITGAV* expression levels in normal pancreatic tissue and pancreatic tumor samples from GEO datasets were compared in graphpad prism 9.3.0. In the Badea (GSE15471) dataset, pairs of normal (*n* = 39) and tumor (*n* = 39) tissue samples were obtained at the time of surgery [26]. In the Moffit (GSE71729) dataset, normal (*n* = 46) and tumor (*n* = 145) tissue samples were obtained from PDAC cases [27]. In the Pei (GSE16515) dataset, normal (*n* = 16) and tumor (*n* = 36) tissue samples were obtained at the time of surgery [28]. In the Sandhu (GSE60980) dataset, normal (*n* = 12) and tumor (*n* = 49) tissue samples were obtained from PDAC cases [29]. In the Yang (GSE62452) dataset, normal (*n* = 61) and tumor (*n* = 69) tissue samples were obtained from PDAC cases [30]. In the Zhang (GSE28735) dataset, matched pairs of normal (*n* = 45) and tumor (*n* = 45) tissue samples were obtained at the time of surgery [31]. *P* values were calculated using a two‐tailed Mann–Whitney test.

### 2.16 Protein expression analysis

Normalized ITGAV expression levels in normal pancreatic tissue and pancreatic tumor samples from the CPTAC‐PDAC patient dataset were compared in graphpad prism 9.3.0. In the CPTAC‐PDAC dataset, normal (*n* = 75) and tumor (*n* = 140) tissue samples were obtained from PDAC cases [32]. *P* values were calculated using a two‐tailed Mann–Whitney test.

### 2.17 Gene set enrichment analysis (GSEA)

GSEA was performed on the TCGA‐PAAD patient dataset by median *ITGAV* expression as previously described [33].

## 3 Results

### 3.1 High *ITGAV* expression in PDAC is associated with poor outcomes

**Fig. 1:**
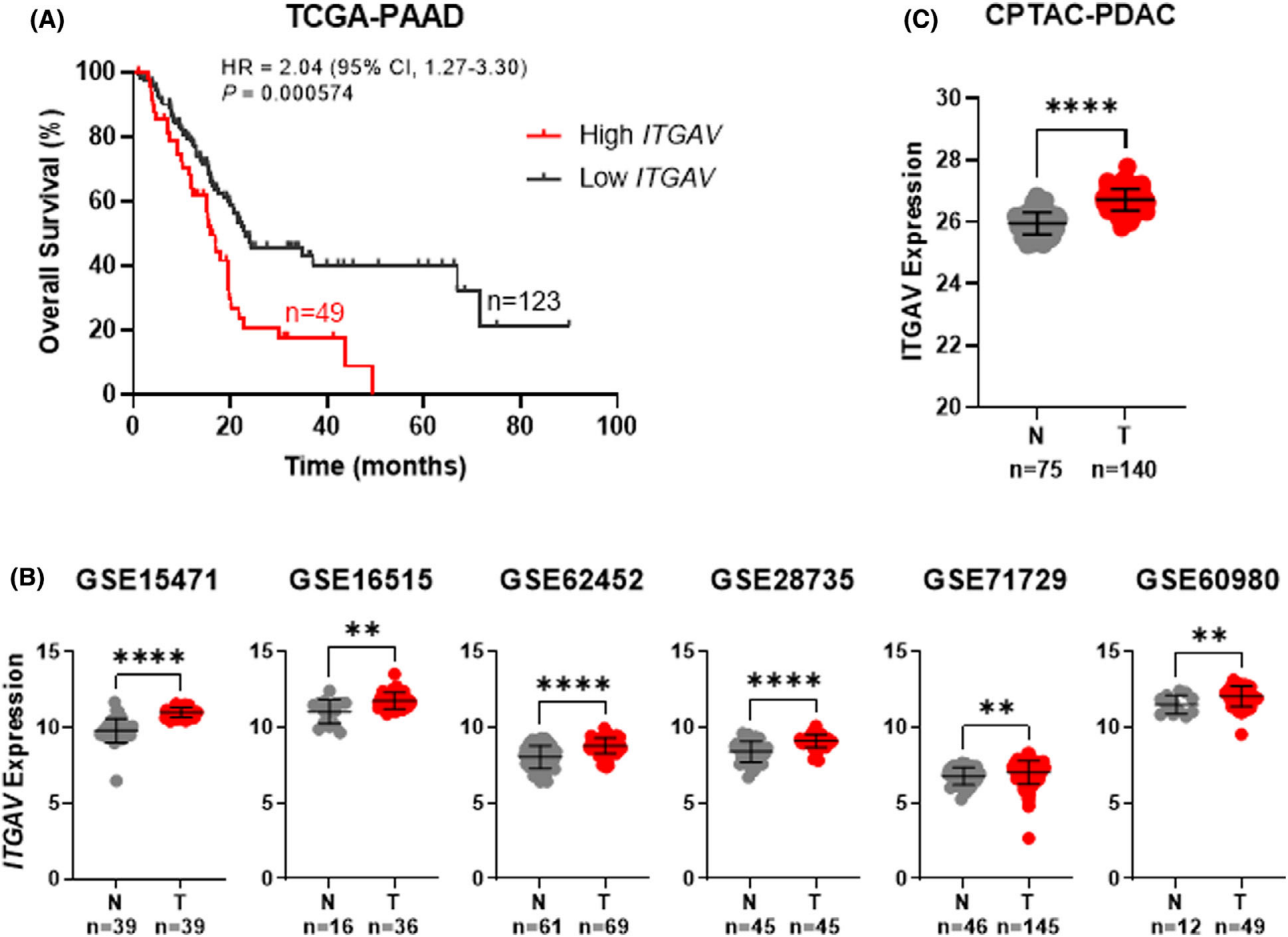
High *ITGAV* expression in pancreatic ductal adenocarcinoma (PDAC) is associated with poor outcomes. (A) High *ITGAV* expression in the TCGA‐PAAD dataset was associated with shorter overall survival and increased risk of death compared to low *ITGAV* expression. (B) Normalized *ITGAV* expression levels are elevated in pancreatic tumor samples (T) compared to normal pancreatic tissue (N) across GEO datasets. Error bars given as mean ± SD. **P < 0.01, ****P < 0.0001 by Mann–Whitney test. (C) Normalized ITGAV expression levels are elevated in pancreatic tumor samples (T) compared to normal pancreatic tissue (N) in the CPTAC‐PDAC dataset. Error bars given as mean ± SD. ****P < 0.0001 by Mann–Whitney test.

Kaplan–Meier survival analyses with data from The Cancer Genome Atlas (TCGA) database [34] revealed that patients with high *ITGAV* expression have a significantly shorter overall survival (*P* = 0.000574) and an increased risk of death (HR = 2.04) compared to patients with low *ITGAV* expression (Fig. 1A). Consistent with these findings, we observed that both the mRNA and protein expression of ITGAV was significantly higher in pancreatic tumor samples compared to normal pancreatic tissue samples across seven independent datasets: GSE15471 (1.14‐fold) [26], GSE16515 (1.04‐fold) [28], GSE62452 (1.10‐fold) [30], GSE28735 (1.09‐fold) [31], GSE71729 (1.05‐fold) [27], GSE60980 (1.06‐fold) [29], and the CPTAC‐PDAC dataset (1.03‐fold) [32] (Fig. 1B,C). Together, these results demonstrate that *ITGAV* is consistently overexpressed, and higher levels are associated with poor outcomes in PDAC.

### 3.2 ITGAV regulates cell proliferation, migration, and invasion in PDAC

**Fig. 2:**
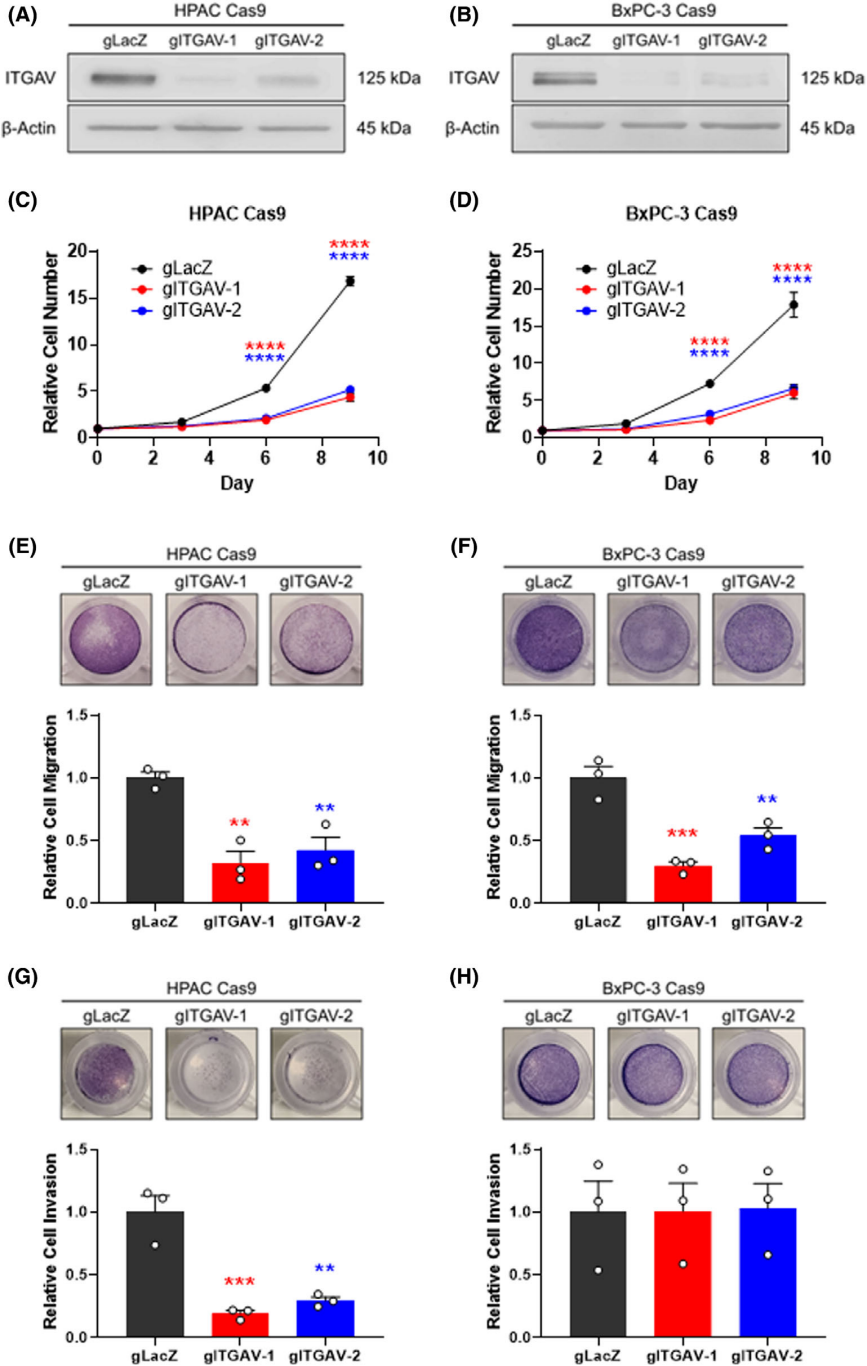
*ITGAV* knockout (KO) inhibits pancreatic ductal adenocarcinoma (PDAC) cell proliferation and migration, but invasion is cell type‐specific. Western blot analysis of ITGAV levels in (A) HPAC and (B) BxPC‐3 Cas9 cells stably transduced with a sgRNA targeting *ITGAV*. Representative image, *n* = 3 independent experiments. Proliferation assay in (C) HPAC and (D) BxPC‐3 Cas9 cells infected with sgRNA‐*LacZ* or sgRNA‐*ITGAV*, showing reduced proliferation upon *ITGAV* KO. Each point represents the mean ± SEM from *n* = 3 independent experiments. ****P < 0.0001 from sgRNA‐*LacZ* by two‐way ANOVA with Dunnett's post hoc. Transwell migration assay in (E) HPAC and (F) BxPC‐3 Cas9 cells infected with sgRNA‐*LacZ* or sgRNA‐*ITGAV*, showing reduced migration upon *ITGAV* KO. Representative images of stained, migrated cells were taken 48 h after seeding. Bars represent mean ± SEM from *n* = 3 independent experiments. **P < 0.01, ***P < 0.001 from sgRNA‐*LacZ* by one‐way ANOVA with Dunnett's post hoc. Transwell invasion assay in (G) HPAC and (H) BxPC‐3 Cas9 cells infected with sgRNA‐*LacZ* or sgRNA‐*ITGAV*, showing reduced invasion upon *ITGAV* KO only in HPAC cells. Representative images of stained, invaded cells were taken 48 h after seeding. Bars represent mean ± SEM from *n* = 3 independent experiments. **P < 0.01, ***P < 0.001 from sgRNA‐*LacZ* by one‐way ANOVA with Dunnett's post hoc.

The functional role of ITGAV in PDAC cells was investigated through experiments measuring proliferation, migration, and invasion. CRISPR/Cas9‐mediated *ITGAV* knockout (KO) in PANC‐1, PK‐1, HPAC, and BxPC‐3 PDAC cell lines (Fig. 2A,B; Fig. S1A,B) resulted in a significant reduction in cell growth as demonstrated by proliferation assays (Fig. 2C,D; Fig. S1C,D). Rescue experiments using HPAC cells transduced with an *ITGAV‐expressing* construct with altered sgRNA binding sites, thus resistant to *ITGAV* KO, demonstrated that growth phenotypes were dependent on *ITGAV* depletion (Fig. S2). *ITGAV* KO also decreased the migratory capacity of all PDAC cell lines in transwell migration assays (Fig. 2E,F; Fig. S1E,F).

The invasive potential of PDAC cells with *ITGAV* KO was also evaluated using transwell invasion assays. However, these assays utilized Matrigel—a matrix composed of extracellular proteins such as laminin, collagen IV, heparin sulfate proteoglycans, and entactin/nidogen—to coat the transwell and mimic the extracellular environment. *ITGAV* KO demonstrated a significant reduction in invasion in HPAC and PANC‐1 cells (Fig. 2G; Fig. S1G). Surprisingly, *ITGAV* KO in BxPC‐3 and PK‐1 cells showed no difference in invasive capacity (Fig. 2H; Fig. S1H). Taken together, these results demonstrate a crucial role for ITGAV in the growth and migratory properties of PDAC cells, while also demonstrating a complex relationship between ITGAV and the invasive potential of different PDAC cell lines.

### 3.3 ITGAV differentially regulates TGF‐β and MAPK/ERK signaling in PDAC

**Fig. 3:**
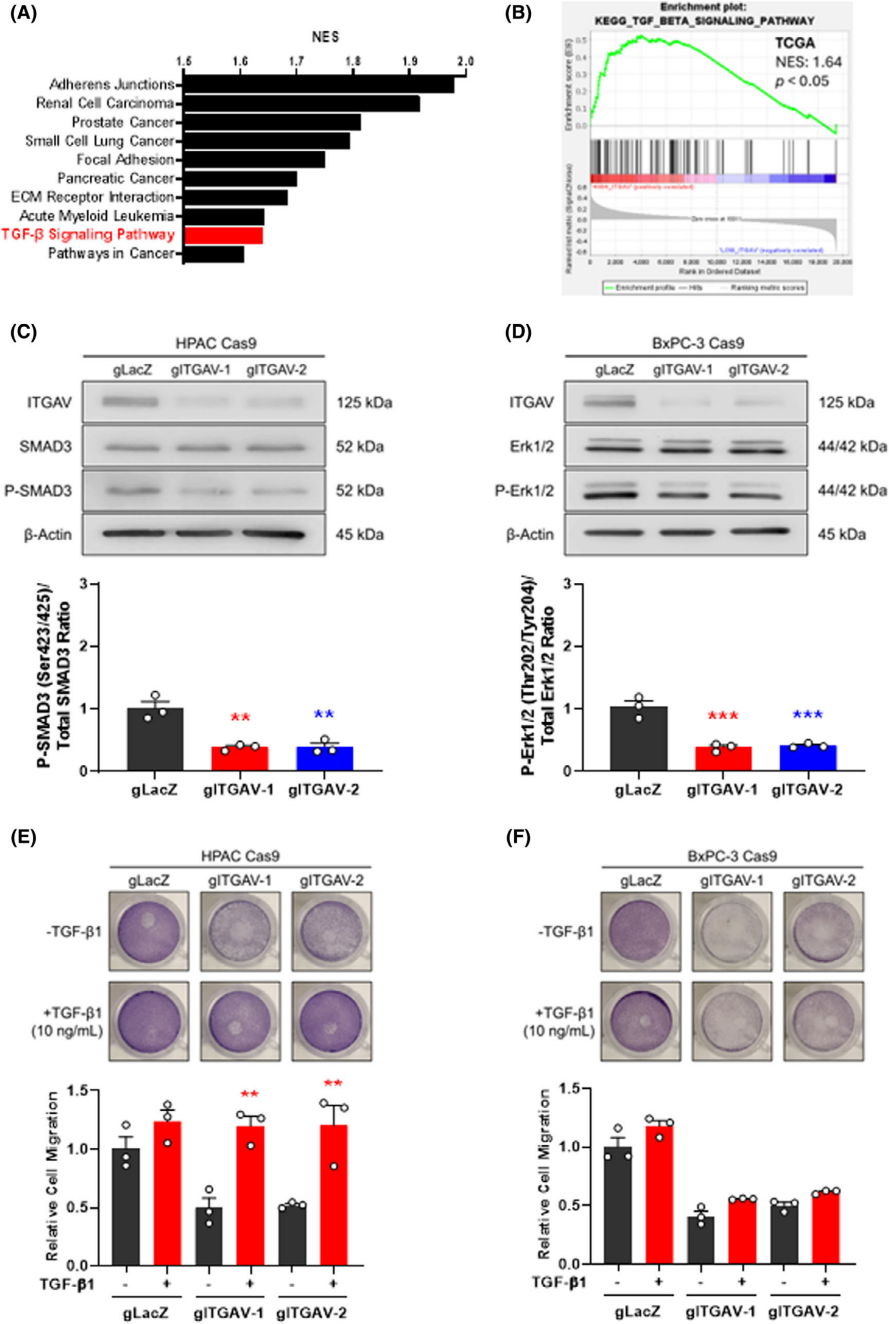
ITGAV can signal through transforming growth factor‐beta (TGF‐β) or mitogen‐activated protein kinase (MAPK)/extracellular signal‐regulated kinase (ERK) pathways in pancreatic ductal adenocarcinoma (PDAC). (A) Top 10 Kyoto Encyclopedia of Genes and Genomes (KEGG) gene sets enriched in *ITGAV* ^*high*^ patients from the TCGA‐PAAD dataset, as determined by Gene Set Enrichment Analysis (GSEA), with the TGF‐β signaling pathway—relevant to both PDAC progression and integrin signaling—highlighted in red. (B) Enrichment plot for KEGG TGF‐β signaling pathway genes enriched in *ITGAV* ^*high*^ patients from the TCGA‐PAAD dataset. (C) Western blot analysis of SMAD3 and phospho‐SMAD3 (Ser423/425) levels in HPAC Cas9 cells infected with sgRNA‐*LacZ* or sgRNA‐*ITGAV*, showing reduced phospho‐SMAD3 levels upon *ITGAV* KO. Representative image, bars represent mean ± SEM from *n* = 3 independent experiments. **P < 0.01 from sgRNA‐*LacZ* by one‐way ANOVA with Dunnett's post hoc. (D) Western blot analysis of Erk1/2 and phospho‐Erk1/2 (Thr202/Tyr204) levels in BxPC‐3 Cas9 cells infected with sgRNA‐*LacZ* or sgRNA‐*ITGAV*, showing reduced phospho‐Erk1/2 upon *ITGAV* KO. Representative image, bars represent mean ± SEM from *n* = 3 independent experiments. ***P < 0.001 from sgRNA‐*LacZ* by one‐way ANOVA with Dunnett's post hoc. Transwell migration assay in (E) HPAC and (F) BxPC‐3 Cas9 cells infected with sgRNA‐*LacZ* or sgRNA‐*ITGAV*, treated with active TGF‐β1 ligand, showing rescued migration only in *ITGAV* KO HPAC cells. Representative images of stained, migrated cells were taken 48 h after seeding. Bars represent mean ± SEM from *n* = 3 independent experiments. **P < 0.01 from –TGF‐β1 condition by one‐way ANOVA with Sidak's post hoc.

To gain a deeper understanding of the biological pathways linked to *ITGAV* expression, gene set enrichment analysis (GSEA) was conducted on gene expression data from *ITGAV* ^*high*^ vs. *ITGAV* ^*low*^ expressing tumors in the TCGA‐PAAD dataset. As expected, adherens junctions and other integrin‐related gene sets, like focal adhesion and ECM receptor interaction, were ranked among the top 10 enriched Kyoto Encyclopedia of Genes and Genomes (KEGG) gene sets in *ITGAV* ^*high*^ patients (Fig. 3A). Notably, the TGF‐β signaling pathway was among the most significantly enriched gene sets in *ITGAV* ^*high*^ patients (NES: 1.64, *P* < 0.05) (Fig. 3A,B; Fig. S3A). These findings are consistent with previous studies reporting that ITGAV regulates TGF‐β signaling by contributing to the conversion of latent TGF‐β ligand to active TGF‐β ligand [35, 36, 37, 38, 39].

TGF‐β signaling is well established to play a critical role in PDAC, as evidenced by the frequent mutations in *SMAD4*, a tumor suppressor gene encoding a transcription factor that is a central mediator of the TGF‐β pathway [40]. *SMAD4* inactivating mutations are present in approximately 50% of PDAC patients and lead to impaired TGF‐β pathway signaling and loss of its tumor suppressive functions [40, 41]. To investigate whether the peculiar results we observed in our invasion assays following *ITGAV* KO in PDAC cell lines are associated with *SMAD4* status, we checked the status of SMAD4 in our PDAC cell line panel by western blot. We observed that SMAD4 protein was present in HPAC and PANC‐1 cells, but undetectable in BxPC‐3 and PK‐1 cells (Fig. S3B). These data revealed that *ITGAV* KO in *SMAD4*‐expressing cells (HPAC and PANC‐1) resulted in impaired invasion, while *SMAD4*‐deficient cells (BxPC‐3 and PK‐1) maintained invasive capacity upon *ITGAV* KO.

To further understand the roles for ITGAV in TGF‐β signaling in PDAC cells, we also examined phosphorylation levels of SMAD3, a key receptor‐activated SMAD protein in the TGF‐β signaling pathway [41]. Western blot analysis revealed a significant decrease in phosphorylated SMAD3 levels in *SMAD4*‐expressing HPAC cells upon *ITGAV* KO (Fig. 3C), whereas no change in phosphorylated SMAD3 was detected in *SMAD4*‐deficient BxPC‐3 cells (Fig. S3C). These findings confirm that ITGAV contributes to TGF‐β signaling in *SMAD4* proficient cells, but not in those where TGF‐β signal transduction is disrupted by *SMAD4* loss. In addition to activating TGF‐β signaling, integrin signaling is also known to influence other signaling pathways, including MAPK/ERK signaling [42, 43]. Notably, when examining MAPK/ERK signaling in PDAC cell lines, we found that *ITGAV* KO led to a decrease in ERK phosphorylation in *SMAD4*‐deficient BxPC‐3 cells (Fig. 3D), but did not affect ERK phosphorylation in *SMAD4*‐expressing HPAC cells (Fig. S3D). These findings indicate that downstream *ITGAV* signaling pathways are influenced by *SMAD4* status in PDAC cells.

Given that ITGAV contributes to TGF‐β1 ligand activation [35, 36, 37, 38, 39], we performed transwell migration assays in *ITGAV* KO cells in the presence of active TGF‐β1 ligand, which would bypass the need for ITGAV function. As expected, active TGF‐β1 rescued the migratory and invasive defects in SMAD4‐expressing HPAC cells upon *ITGAV* KO (Fig. 3E; Fig. S3E). However, active TGF‐β1 was unable to restore the migration defect observed in SMAD4‐deficient BxPC‐3 cells (Fig. 3F). Together, these findings identify an intricate interplay between ITGAV, TGF‐β activation, MAPK/ERK signaling, and invasion in PDAC, underscoring the complexity of integrin‐mediated pathways in PDAC progression.

### 3.4 The ITGAV‐SMAD4 axis modulates *MMP9* expression and invasion in PDAC

**Fig. 4:**
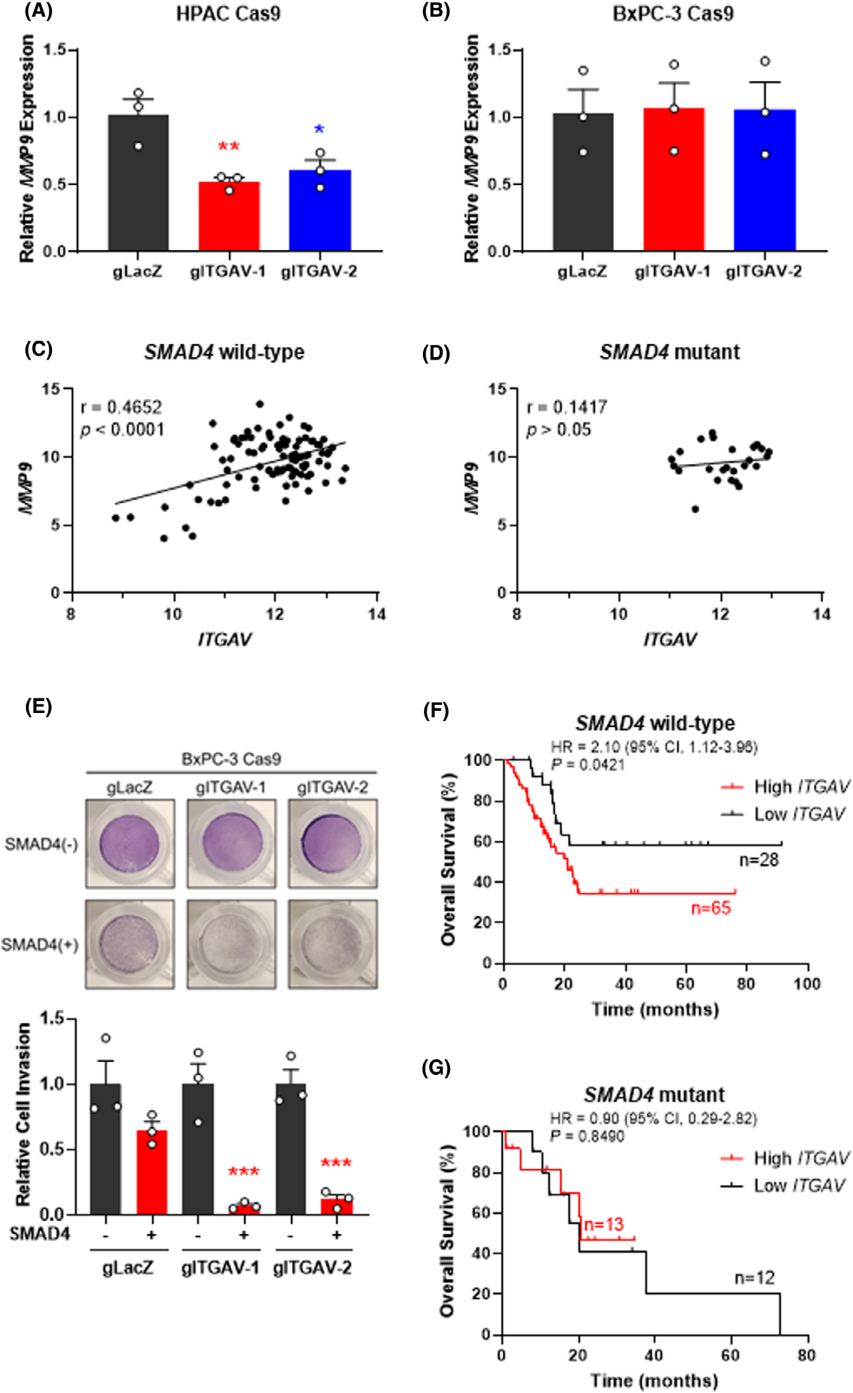
ITGAV‐SMAD4 axis modulates *matrix metalloproteinase* 9 (*MMP9*) expression and invasion in pancreatic ductal adenocarcinoma (PDAC). Quantitative polymerase chain reaction (qPCR) of *MMP9* in (A) HPAC and (B) BxPC‐3 Cas9 cells infected with sgRNA‐*LacZ* or sgRNA‐*ITGAV*, showing reduced *MMP9* transcript levels only in *ITGAV KO* HPAC cells. Bars represent mean ± SEM from *n* = 3 independent experiments. *P < 0.05, **P < 0.01 from sgRNA‐*LacZ* by one‐way ANOVA with Dunnett's post hoc. Pearson correlation analysis comparing the expression of *ITGAV* and *MMP9* in (C) *SMAD4* wild‐type and (D) *SMAD4* mutant PDAC patients in the TCGA‐PAAD dataset, showing a strong positive correlation in *SMAD4* wild‐type patients. (E) Transwell invasion assay in *SMAD4*‐null and *SMAD4*‐expressing BxPC‐3 Cas9 cells infected with sgRNA‐*LacZ* or sgRNA‐*ITGAV*, showing reduced invasion only in *SMAD4*‐expressing *ITGAV* KO cells. Representative images of stained, invaded cells were taken 48 h after seeding. Bars represent mean ± SEM from *n* = 3 independent experiments. ***P < 0.001 from –SMAD4 condition by one‐way ANOVA with Sidak's post hoc. Kaplan–Meier plot for (F) *SMAD4* wild‐type and (G) *SMAD4* mutant PDAC patients comparing *ITGAV* ^high^ vs. *ITGAV* ^low^ expression, showing shorter overall survival and an increased risk of death in *SMAD4* wild‐type patients with high *ITGAV* expression.

Given the critical role of integrins in regulating the ECM, we also investigated how ITGAV influences the expression of matrix metalloproteinases (MMPs)—a large family of calcium‐dependent, zinc‐containing endopeptidases involved in tissue remodeling and ECM degradation [44]. Among the MMPs, MMP9 is particularly well‐studied in cancer due to its significant role in ECM remodeling and tumor invasion [45]. Thus, we first investigated whether *ITGAV* KO affected *MMP9* expression, as a mechanism to explain how ITGAV may influence the invasive behavior of PDAC cells. Analysis of *MMP9* expression revealed that *ITGAV* KO decreased *MMP9* transcript levels in HPAC cells (Fig. 4A) but did not affect *MMP9* levels in BxPC‐3 cells (Fig. 4B). Consistent with this, we identified a strong correlation between *ITGAV* and *MMP9* expression in PDAC patients with wild‐type *SMAD4* status (*r* = 0.4652, *P* < 0.0001) (Fig. 4C), whereas patients with mutant *SMAD4* status demonstrated no correlation between *ITGAV* and *MMP9* expression (*r* = 0.1417, *P* > 0.05) (Fig. 4D).

To corroborate these findings in our cell models, we rescued wild‐type *SMAD4* expression in *SMAD4*‐deficient BxPC‐3 cells (Fig. S4A) and evaluated their invasive potential, TGF‐β signaling activation, and *MMP9* expression upon *ITGAV* KO. When examining TGF‐β signal transduction, we found that *ITGAV* KO led to a decrease in SMAD3 phosphorylation in *SMAD4*‐rescued BxPC‐3 cells but not in control *SMAD4*‐null BxPC‐3 cells (Fig. S4B). Moreover, *ITGAV* KO resulted in a decrease in *MMP9* expression in *SMAD4*‐rescued *ITGAV* KO cells but not in *SMAD4*‐null cells (Fig. S4C). Finally, in transwell invasion assays, BxPC‐3 cells ectopically expressing wild‐type *SMAD4* demonstrated decreased invasive capabilities upon *ITGAV* loss, whereas control *SMAD4*‐null cells exhibited no change (Fig. 4E).

To assess the potential clinical significance of these findings, we reanalyzed the TCGA‐PAAD survival data based on *ITGAV* expression, this time taking *SMAD4* status into account. In this reanalysis, we observed that in *SMAD4* wild‐type patients, high *ITGAV* expression was associated with decreased patient survival (*P* = 0.0421) and increased risk of death (HR = 2.10) (Fig. 4F). Conversely, in *SMAD4* mutant patients, *ITGAV* expression was unable to stratify poor and favorable survival (*P* = 0.8490, HR = 0.90) (Fig. 4G). Altogether, these results highlight a critical role of ITGAV in regulating *MMP9* expression, the invasive potential of PDAC cells, and overall PDAC patient survival in a SMAD4‐dependent manner.

### 3.5 αV integrin inhibition with GLPG0187 mimics *ITGAV* KO in PDAC

**Fig. 5:**
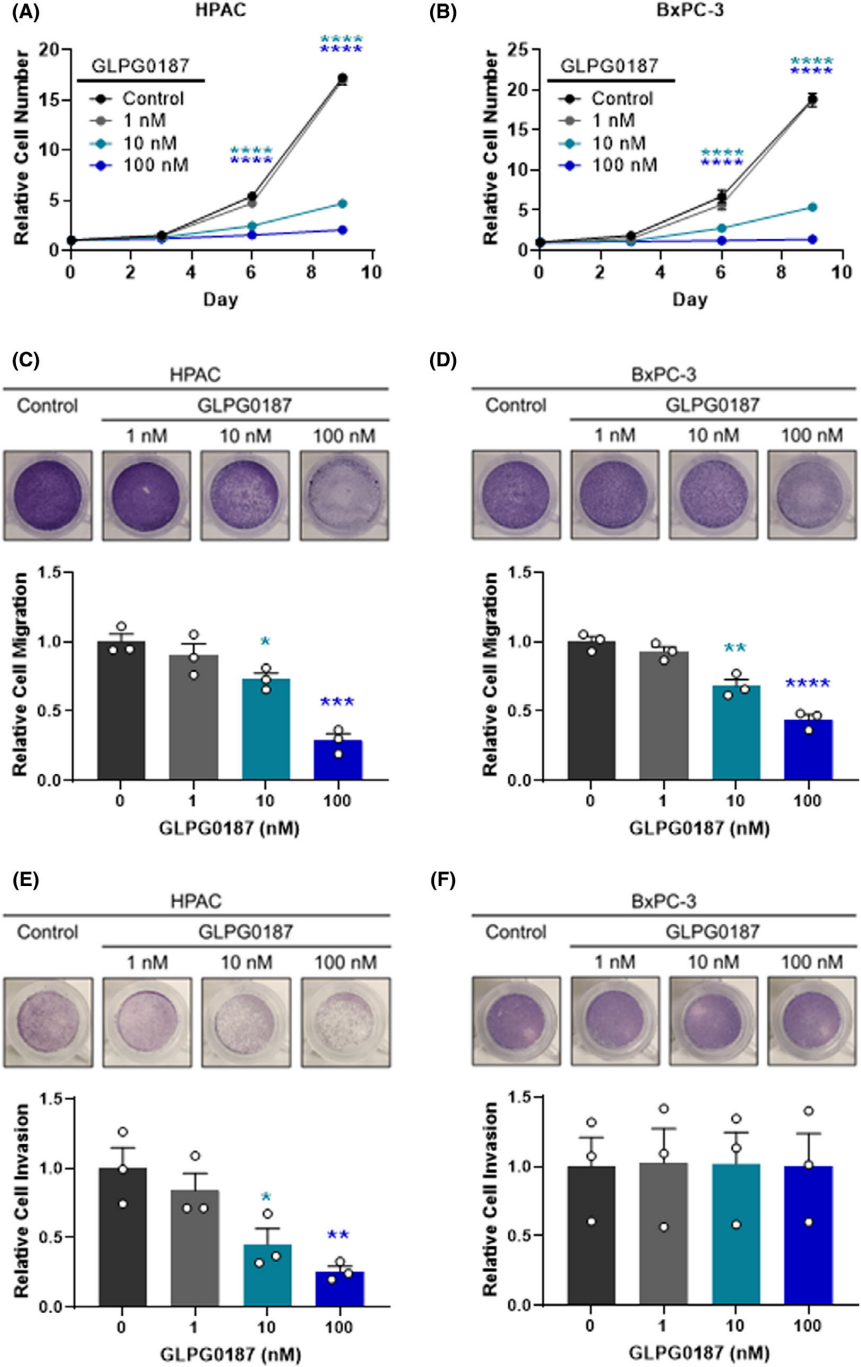
αV integrin inhibition suppresses pancreatic ductal adenocarcinoma (PDAC) cell proliferation and migration, whereas invasion is SMAD4‐dependent. Proliferation assay in (A) HPAC and (B) BxPC‐3 cells treated with GLPG0187, showing a dose‐dependent decrease in proliferation. Each point represents the mean ± SEM from *n* = 3 independent experiments. ****P < 0.0001 from control by two‐way ANOVA with Dunnett's post hoc. Transwell migration assay in (C) HPAC and (D) BxPC‐3 cells treated with GLPG0187, showing a dose‐dependent decrease in migration. Representative images of stained, migrated cells were taken 48 h after seeding. Bars represent mean ± SEM from *n* = 3 independent experiments. *P < 0.05, **P < 0.01, ***P < 0.001, ****P < 0.0001 from control by one‐way ANOVA with Dunnett's post hoc. Transwell invasion assay in (E) HPAC and (F) BxPC‐3 cells treated with GLPG0187, showing a dose‐dependent decrease in invasion only in HPAC cells. Representative images of stained, invaded cells were taken 48 h after seeding. Bars represent mean ± SEM from *n* = 3 independent experiments. *P < 0.05, **P < 0.01 from control by one‐way ANOVA with Dunnett's post hoc.

Given the availability of integrin inhibitors, we evaluated the effects of pharmacological inhibition of ITGAV on cell proliferation, migration, and invasion. GLPG0187 is a broad‐spectrum integrin antagonist that inhibits all known αV integrin receptors (αVβ1, αVβ3, αVβ5, αVβ6, αVβ8) with high affinity and α5β1 with low affinity [46, 47, 48, 49]. Similar to phenotypes in *ITGAV* KO PDAC cells, treatment with GLPG0187 decreased proliferation and migration in all PDAC cell lines (Fig. 5A–D; Fig. S5A–D), but only impaired invasion in SMAD4‐expressing HPAC and PANC‐1 cells (Fig. 5E,F; Fig. S5E,F). These findings indicate that GLPG0187‐mediated inhibition of invasion is SMAD4‐dependent. Overall, the results show that pharmacologic blockade of αV integrins with GLPG0187 recapitulates the effects of ITGAV genetic knockout in PDAC cells *in vitro*.

## 4 Discussion

PDAC is a highly lethal cancer with limited treatment options, especially for patients with advanced disease [50]. While targeting αV integrins, including ITGAV, has shown promise in preclinical studies across various cancers [51, 52, 53, 54, 55], clinical trials in pancreatic cancer, head and neck cancer, lung cancer, melanoma, and glioblastoma using αV integrin inhibitors have been largely disappointing [16, 17, 18, 19, 20, 21]. Our study was geared at investigating ITGAV function in PDAC progression, aiming to identify effective PDAC subtype‐specific therapeutic strategies. ITGAV emerged as a critical regulator of molecular pathways driving PDAC progression, particularly in its interaction with TGF‐β signaling and ECM remodeling pathways. Notably, ITGAV modulates distinct downstream signaling pathways contingent on *SMAD4* status: in *SMAD4*‐proficient cells, it activates TGF‐β signaling to enhance invasion and *MMP9* expression, while in *SMAD4*‐deficient cells, it signals primarily through the MAPK/ERK pathway.

Consistent overexpression of *ITGAV* across multiple independent datasets, and its correlation with poor patient survival in TCGA‐PAAD, suggest that *ITGAV* expression is a putative biomarker for a more aggressive PDAC phenotype. Accordingly, CRISPR‐mediated KO of *ITGAV* led to decreased proliferation and migration across all PDAC cell lines tested, underscoring the broad functional importance of this integrin in tumor cell growth and motility. However, *ITGAV* loss only impaired the invasive capacity of *SMAD4*‐expressing PDAC lines (HPAC and PANC‐1), pointing to a requirement for intact SMAD4‐mediated signaling in the execution of ITGAV‐dependent invasive programs. Notably, the disconnect between migration and invasion observed in BxPC‐3 and PK‐1 cells is unusual, but has been previously reported in post‐EMT prostate cancer and human mammary epithelial cell lines [56].

GSEA of TCGA‐PAAD tumors with high *ITGAV* expression revealed enrichment in canonical integrin signaling pathways, including focal adhesion, ECM receptor interaction, and adherens junctions. Notably, high *ITGAV* expression was also linked to TGF‐β Signaling, supporting previous findings that αV integrins contribute to the mechanical activation of latent TGF‐β ligands [35, 36, 37, 38, 39]. This was further validated by our observations that *ITGAV* KO reduces SMAD3 phosphorylation specifically in *SMAD4*‐proficient cells. Once activated by ITGAV, TGF‐β ligand binds to its receptor complex, leading to the activation of TGF‐β receptor I (TβRI) kinase. TβRI then phosphorylates SMAD3 at serine residues 423 and 425—a critical step for SMAD3 activation. Activated SMAD3 forms a complex with SMAD4 and translocates to the nucleus to regulate target gene expression [57]. These results align with established models showing that αV integrins facilitate the mechanical activation of latent TGF‐β, triggering downstream SMAD2/3 signaling [35, 36, 37, 38, 39]. However, in *SMAD4‐*null PDAC cells, this pathway is disrupted, leading to the loss of ITGAV function role in canonical TGF‐β signaling.

Further dissecting this relationship, we found that ITGAV regulates the expression of *MMP9*, a key effector of ECM degradation and invasion, in a SMAD4‐dependent manner [44, 45]. This was validated both *in vitro*—where *ITGAV* KO suppressed *MMP9* only in *SMAD4*‐proficient contexts—and *in silico* in TCGA data, where a strong correlation between *ITGAV* and *MMP9* expression was observed only in *SMAD4* wild‐type tumors. Notably, rescue of *SMAD4* expression in BxPC‐3 cells restored TGF‐β signal transduction as shown by SMAD3 phosphorylation and *MMP9* expression. Furthermore, *SMAD4* expression rescued ITGAV‐dependent invasive behavior, providing evidence that the ITGAV–SMAD4–MMP9 signaling axis is a key mediator of ECM remodeling and invasion in PDAC.

Interestingly, our data also demonstrate that ITGAV may signal through alternative pathways in the absence of *SMAD4*. In *SMAD4*‐null BxPC‐3 cells, *ITGAV* KO reduced ERK phosphorylation, indicating an alternate signaling pathway and potential compensatory role for MAPK/ERK signaling in supporting tumor cell motility when TGF‐β/SMAD signaling is impaired [58, 59]. Similar findings have been observed in TGF‐β1‐induced autophagy, with TGF‐β and MAPK/ERK signaling leading to different effects on tumor growth and metastasis [60]. This underscores the plasticity of integrin‐mediated signaling and highlights the importance of tumor genotype in shaping downstream signal transduction.

Therapeutically, inhibition of αV integrins using GLPG0187 recapitulated the effects of *ITGAV* genetic ablation, including SMAD4‐dependent blockade of invasion and broad suppression of proliferation and migration. These findings establish GLPG0187 as a candidate therapeutic that may be selectively effective in PDAC tumors with intact SMAD4 signaling. Accordingly, previous work by Hezel and colleagues demonstrated that αVβ6 and TGF‐β act in a common SMAD4‐dependent pathway, with mouse models lacking *Smad4* showing no benefit to αVβ6 inhibition [61]. PDAC patient survival analysis further supports this, showing that high *ITGAV* expression is prognostically relevant only in *SMAD4* wild‐type patients, reinforcing the need for genetic stratification in future clinical applications of integrin inhibitors.

## 5 Conclusions

In summary, our study delineates a mechanistic framework wherein ITGAV promotes PDAC progression by modulating TGF‐β activation and MMP9‐mediated invasion in a SMAD4‐dependent manner. These findings suggest that *ITGAV* expression, in the context of *SMAD4* status, may serve as both a prognostic biomarker and a molecularly defined therapeutic target in PDAC.

## Conflict of interest

The authors declare no conflict of interest.

## Author contributions

DKCL was involved in conceptualization, methodology, software, validation, formal analysis, investigation, data curation, visualization, project administration, writing—original draft, writing—review and editing. KC, RL, XL, DL, GTS, JTSC, CMPM, and LT performed formal analysis and investigation. LS was involved in conceptualization, investigation, resources, supervision, project administration, funding acquisition, writing—original draft, writing—review and editing.

## Peer review

The peer review history for this article is available at https://www.webofscience.com/api/gateway/wos/peer‐review/10.1002/1878‐0261.70080.

## Supporting information


**Fig. S1.** Related to Fig. 2. *ITGAV* knockout (KO) phenotypes supporting information. Western blot analysis of ITGAV levels in (A) PANC‐1 and (B) PK‐1 Cas9 cells stably transduced with a sgRNA targeting *ITGAV*. Representative image, *n* = 3 independent experiments. Proliferation assay in (C) PANC‐1 and (D) PK‐1 Cas9 cells infected with sgRNA‐*LacZ* or sgRNA‐*ITGAV*, showing reduced proliferation upon *ITGAV* KO. Each point represents the mean ± SEM from *n* = 3 independent experiments. *P < 0.05, **P < 0.01, ***P < 0.001, ****P < 0.0001 from sgRNA‐*LacZ* by Two‐Way ANOVA with Dunnett's Post‐Hoc. Transwell migration assay in (E) PANC‐1 and (F) PK‐1 Cas9 cells infected with sgRNA‐*LacZ* or sgRNA‐*ITGAV*, showing reduced migration upon *ITGAV* KO. Representative images of stained, migrated cells were taken 48 h after seeding. Bars represent mean ± SEM from *n* = 3 independent experiments. *P < 0.05, **P < 0.01, ***P < 0.001 from sgRNA‐*LacZ* by One‐Way ANOVA with Dunnett's Post‐Hoc. Transwell invasion assay in (G) PANC‐1 and (H) PK‐1 Cas9 cells infected with sgRNA‐*LacZ* or sgRNA‐*ITGAV*, showing reduced invasion upon *ITGAV* KO only in PANC‐1 cells. Representative images of stained, invaded cells were taken 48 h after seeding. Bars represent mean ± SEM from *n* = 3 independent experiments. **P < 0.01, ***P < 0.001 from sgRNA‐*LacZ* by One‐Way ANOVA with Dunnett's Post‐Hoc.

**Fig. S2.** Related to Fig. 2. ITGAV rescue experiments. (A) Representative images of HPAC cells stably transduced with wild‐type *ITGAV* or sgRNA‐resistant mutant *ITGAV*, and infected with either Cas9‐sgLacZ or Cas9‐sgITGAV‐1 lentivirus. (B) Proliferation assay in wild‐type and sgRNA‐resistant mutant *ITGAV* HPAC cells infected with Cas9‐sgLacZ or Cas9‐sgITGAV‐1, showing reduced proliferation only in wild‐type ITGAV cells. Each point represents the mean ± SD from *n* = 3 independent experiments. ***P < 0.001 by Two‐Way ANOVA with Dunnett's Post‐Hoc.

**Fig. S3.** Related to Fig. 3. ITGAV differential signaling supporting information. (A) Heatmap of KEGG TGF‐β signaling pathway genes enriched in *ITGAV* ^*high*^ patients from the TCGA‐PAAD dataset (upregulated in red, downregulated in blue). (B) Western blot analysis of SMAD4 levels across a panel of four human PDAC cell lines (BxPC‐3, HPAC, PANC‐1, and PK‐1). (C) Western blot analysis of SMAD3 and phospho‐SMAD3 (Ser423/425) levels in BxPC‐3 Cas9 cells infected with sgRNA‐*LacZ* or sgRNA‐*ITGAV*, showing no change in phospho‐SMAD3 levels upon *ITGAV* KO. Representative image, bars represent mean ± SEM from *n* = 3 independent experiments. (D) Western blot analysis of Erk1/2 and phospho‐Erk1/2 (Thr202/Tyr204) levels in HPAC Cas9 cells infected with sgRNA‐*LacZ* or sgRNA‐*ITGAV*, showing no change in phospho‐Erk1/2 levels upon *ITGAV KO*. Representative image, bars represent mean ± SEM from *n* = 3 independent experiments. (E) Transwell invasion assay in HPAC Cas9 cells infected with sgRNA‐*LacZ* or sgRNA‐*ITGAV*, treated with active TGF‐β1 ligand, showing rescued invasion in *ITGAV* KO HPAC cells. Representative images of stained, invaded cells were taken 48 h after seeding. Bars represent mean ± SEM from *n* = 3 independent experiments. ****P < 0.0001 from ‐TGF‐β1 condition by One‐Way ANOVA with Sidak's Post‐Hoc.

**Fig. S4.** Related to Fig. 4. ITGAV‐SMAD4 axis supporting information. (A) Western blot analysis of SMAD4 levels in BxPC‐3 cells stably transduced with a FLAG‐tagged *SMAD4* cDNA. (B) Western blot analysis of SMAD3 and phospho‐SMAD3 (Ser423/425) levels in SMAD4‐null and SMAD4‐expressing BxPC‐3 Cas9 cells infected with sgRNA‐*LacZ* or sgRNA‐*ITGAV*, showing reduced phospho‐SMAD3 levels in SMAD4‐expressing *ITGAV* KO cells. Representative image, bars represent mean ± SEM from *n* = 3 independent experiments. *P < 0.05 from ‐SMAD4 condition by One‐Way ANOVA with Sidak's Post‐Hoc. (C) Quantitative polymerase chain reaction (qPCR) of *MMP9* in SMAD4‐null and SMAD4‐expressing BxPC‐3 Cas9 cells infected with sgRNA‐*LacZ* or sgRNA‐*ITGAV*, showing reduced *MMP9* transcript levels upon *ITGAV* KO in SMAD4‐expressing BxPC‐3 cells. Bars represent mean ± SEM from *n* = 3 independent experiments. *P < 0.05 from ‐SMAD4 condition by One‐Way ANOVA with Sidak's Post‐Hoc.

**Fig. S5.** Related to Fig. 5. αV integrin inhibition phenotypes supporting information. Proliferation assay in (A) PANC‐1 and (B) PK‐1 cells treated with GLPG0187, showing a dose‐dependent decrease in proliferation. Each point represents the mean ± SEM from *n* = 3 independent experiments. *P < 0.05, **P < 0.01, ***P < 0.001, ****P < 0.0001 from Control by Two‐Way ANOVA with Dunnett's Post‐Hoc. Transwell migration assay in (C) PANC‐1 and (D) PK‐1 cells treated with GLPG0187, showing a dose‐dependent decrease in migration. Representative images of stained, migrated cells were taken 48 h after seeding. Bars represent mean ± SEM from *n* = 3 independent experiments. **P < 0.01, ***P < 0.001, ****P < 0.0001 from Control by One‐Way ANOVA with Dunnett's Post‐Hoc. Transwell invasion assay in (E) PANC‐1 and (F) PK‐1 cells treated with GLPG0187, showing a dose‐dependent decrease in invasion only in PK‐1 cells. Representative images of stained, invaded cells were taken 48 h after seeding. Bars represent mean ± SEM from *n* = 3 independent experiments. *P < 0.05, **P < 0.01 from Control by One‐Way ANOVA with Dunnett's Post‐Hoc.

## Data Availability

The data that support the findings of this study are available in the GDC data portal (https://portal.gdc.cancer.gov/), GEO database (https://www.ncbi.nlm.nih.gov/geo/), and PDC data portal (https://pdc.cancer.gov/pdc/).
