# Supplementary figures and images for: ITGAV and SMAD4 influence the progression and clinical outcome of pancreatic ductal adenocarcinoma
Source: Mol Oncol. 2025 Jul 30;19(11):3342–59. doi: 10.1002/1878-0261.70080 (PMC12591305; doi:10.1002/1878-0261.70080)

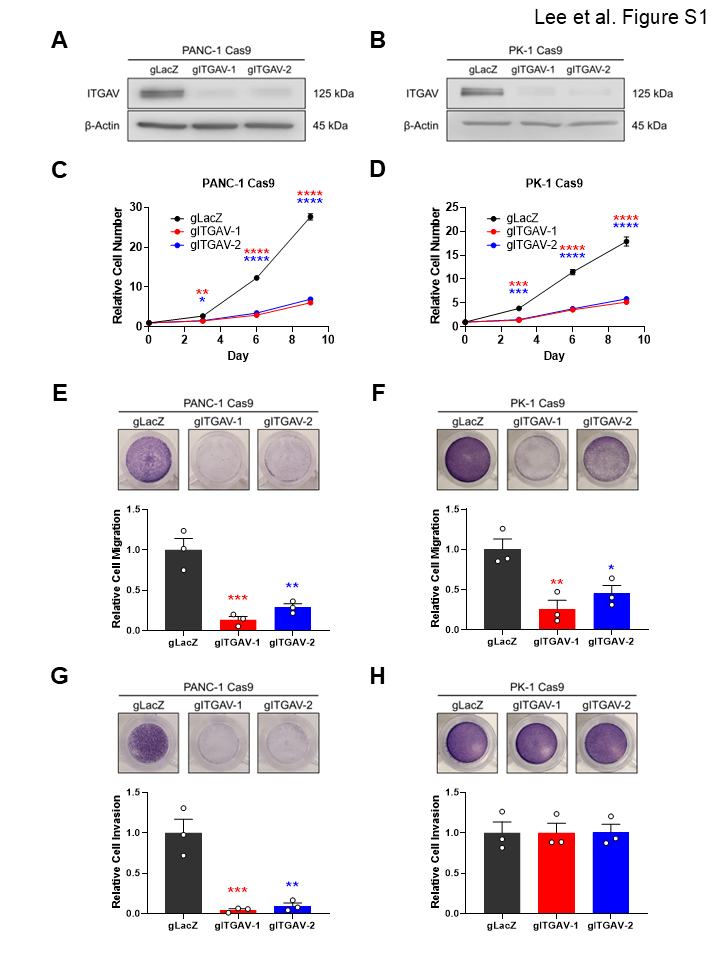

Supplement: Supplementary file 1 — Fig. S1. Related to Fig. 2. ITGAV knockout (KO) phenotypes supporting information. Western blot analysis of ITGAV levels in (A) PANC‐1 and (B) PK‐1 Cas9 cells stably transduced with a sgRNA targeting ITGAV. Representative image, n = 3 independent experiments. Proliferation assay in (C) PANC‐1 and (D) PK‐1 Cas9 cells infected with sgRNA‐LacZ or sgRNA‐ITGAV, showing reduced proliferation upon ITGAV KO. Each point represents the mean ± SEM from n = 3 independent experiments. *P < 0.05, **P < 0.01, ***P < 0.001, ****P < 0.0001 from sgRNA‐LacZ by Two‐Way ANOVA with Dunnett's Post‐Hoc. Transwell migration assay in (E) PANC‐1 and (F) PK‐1 Cas9 cells infected with sgRNA‐LacZ or sgRNA‐ITGAV, showing reduced migration upon ITGAV KO. Representative images of stained, migrated cells were taken 48 h after seeding. Bars represent mean ± SEM from n = 3 independent experiments. *P < 0.05, **P < 0.01, ***P < 0.001 from sgRNA‐LacZ by One‐Way ANOVA with Dunnett's Post‐Hoc. Transwell invasion assay in (G) PANC‐1 and (H) PK‐1 Cas9 cells infected with sgRNA‐LacZ or sgRNA‐ITGAV, showing reduced invasion upon ITGAV KO only in PANC‐1 cells. Representative images of stained, invaded cells were taken 48 h after seeding. Bars represent mean ± SEM from n = 3 independent experiments. **P < 0.01, ***P < 0.001 from sgRNA‐LacZ by One‐Way ANOVA with Dunnett's Post‐Hoc. [file MOL2-19-3342-s001.tiff]

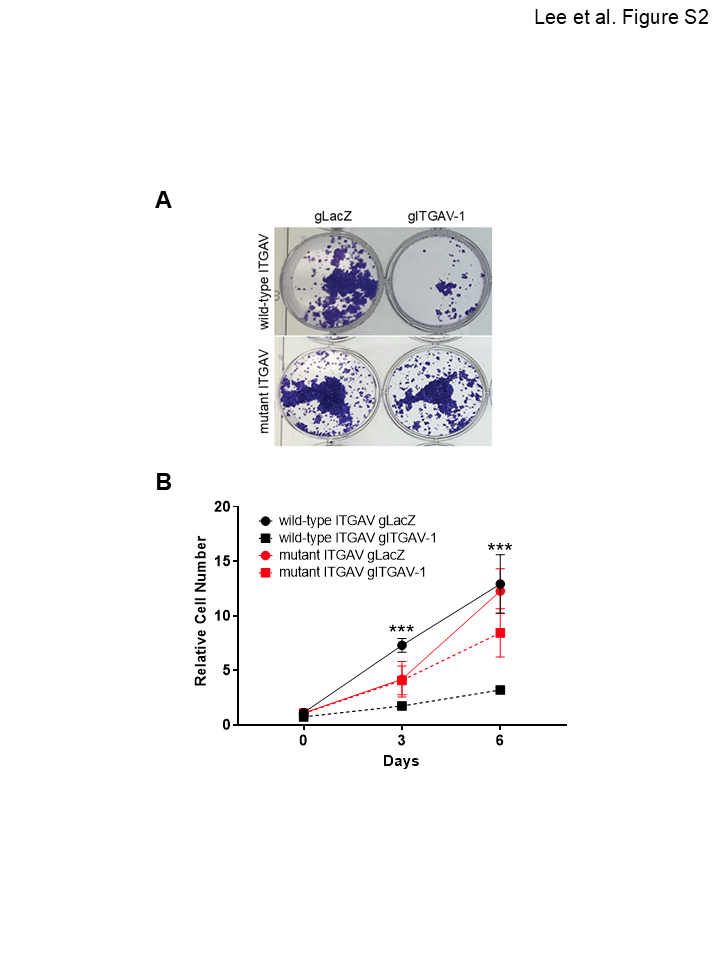

Supplement: Supplementary file 2 — Fig. S2. Related to Fig. 2. ITGAV rescue experiments. (A) Representative images of HPAC cells stably transduced with wild‐type ITGAV or sgRNA‐resistant mutant ITGAV, and infected with either Cas9‐sgLacZ or Cas9‐sgITGAV‐1 lentivirus. (B) Proliferation assay in wild‐type and sgRNA‐resistant mutant ITGAV HPAC cells infected with Cas9‐sgLacZ or Cas9‐sgITGAV‐1, showing reduced proliferation only in wild‐type ITGAV cells. Each point represents the mean ± SD from n = 3 independent experiments. ***P < 0.001 by Two‐Way ANOVA with Dunnett's Post‐Hoc. [file MOL2-19-3342-s002.tiff]

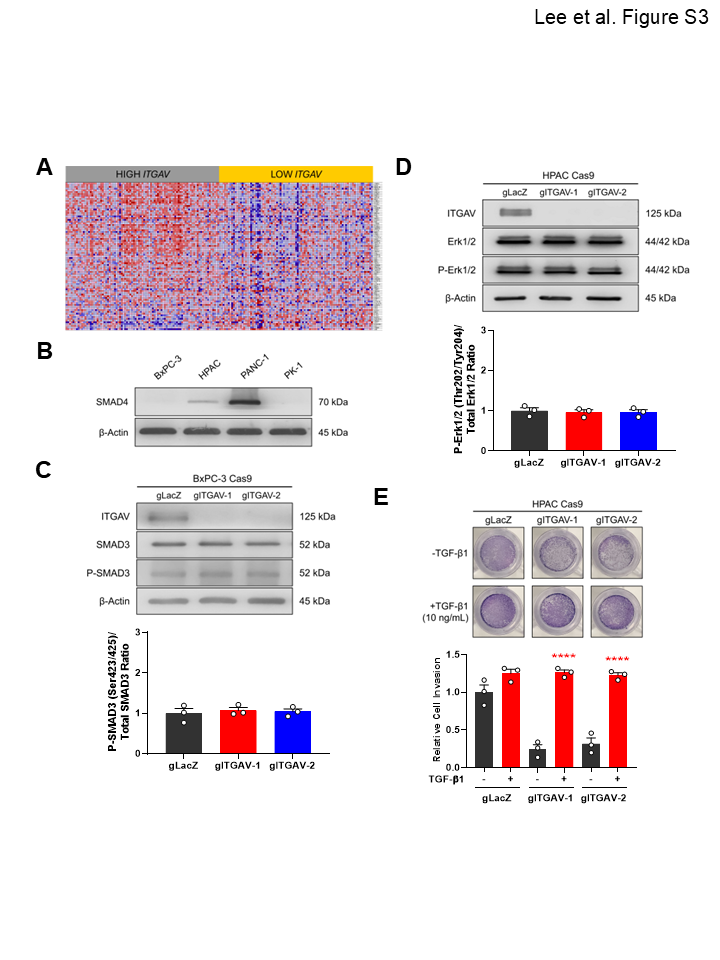

Supplement: Supplementary file 3 — Fig. S3. Related to Fig. 3. ITGAV differential signaling supporting information. (A) Heatmap of KEGG TGF‐β signaling pathway genes enriched in ITGAV high patients from the TCGA‐PAAD dataset (upregulated in red, downregulated in blue). (B) Western blot analysis of SMAD4 levels across a panel of four human PDAC cell lines (BxPC‐3, HPAC, PANC‐1, and PK‐1). (C) Western blot analysis of SMAD3 and phospho‐SMAD3 (Ser423/425) levels in BxPC‐3 Cas9 cells infected with sgRNA‐LacZ or sgRNA‐ITGAV, showing no change in phospho‐SMAD3 levels upon ITGAV KO. Representative image, bars represent mean ± SEM from n = 3 independent experiments. (D) Western blot analysis of Erk1/2 and phospho‐Erk1/2 (Thr202/Tyr204) levels in HPAC Cas9 cells infected with sgRNA‐LacZ or sgRNA‐ITGAV, showing no change in phospho‐Erk1/2 levels upon ITGAV KO. Representative image, bars represent mean ± SEM from n = 3 independent experiments. (E) Transwell invasion assay in HPAC Cas9 cells infected with sgRNA‐LacZ or sgRNA‐ITGAV, treated with active TGF‐β1 ligand, showing rescued invasion in ITGAV KO HPAC cells. Representative images of stained, invaded cells were taken 48 h after seeding. Bars represent mean ± SEM from n = 3 independent experiments. ****P < 0.0001 from ‐TGF‐β1 condition by One‐Way ANOVA with Sidak's Post‐Hoc. [file MOL2-19-3342-s004.tiff]

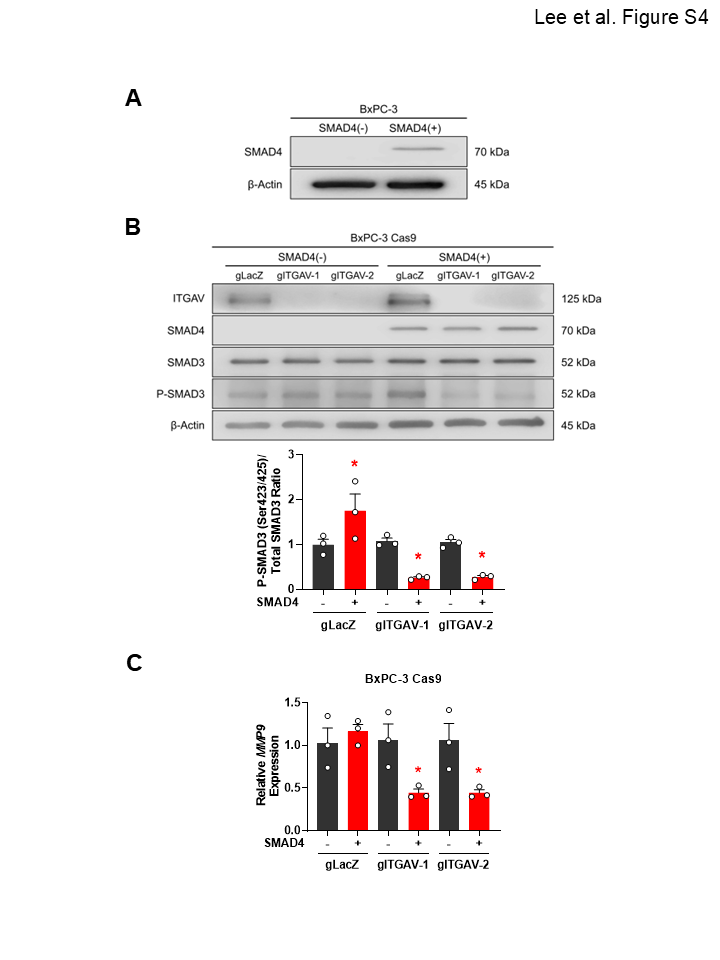

Supplement: Supplementary file 4 — Fig. S4. Related to Fig. 4. ITGAV‐SMAD4 axis supporting information. (A) Western blot analysis of SMAD4 levels in BxPC‐3 cells stably transduced with a FLAG‐tagged SMAD4 cDNA. (B) Western blot analysis of SMAD3 and phospho‐SMAD3 (Ser423/425) levels in SMAD4‐null and SMAD4‐expressing BxPC‐3 Cas9 cells infected with sgRNA‐LacZ or sgRNA‐ITGAV, showing reduced phospho‐SMAD3 levels in SMAD4‐expressing ITGAV KO cells. Representative image, bars represent mean ± SEM from n = 3 independent experiments. *P < 0.05 from ‐SMAD4 condition by One‐Way ANOVA with Sidak's Post‐Hoc. (C) Quantitative polymerase chain reaction (qPCR) of MMP9 in SMAD4‐null and SMAD4‐expressing BxPC‐3 Cas9 cells infected with sgRNA‐LacZ or sgRNA‐ITGAV, showing reduced MMP9 transcript levels upon ITGAV KO in SMAD4‐expressing BxPC‐3 cells. Bars represent mean ± SEM from n = 3 independent experiments. *P < 0.05 from ‐SMAD4 condition by One‐Way ANOVA with Sidak's Post‐Hoc. [file MOL2-19-3342-s005.tiff]

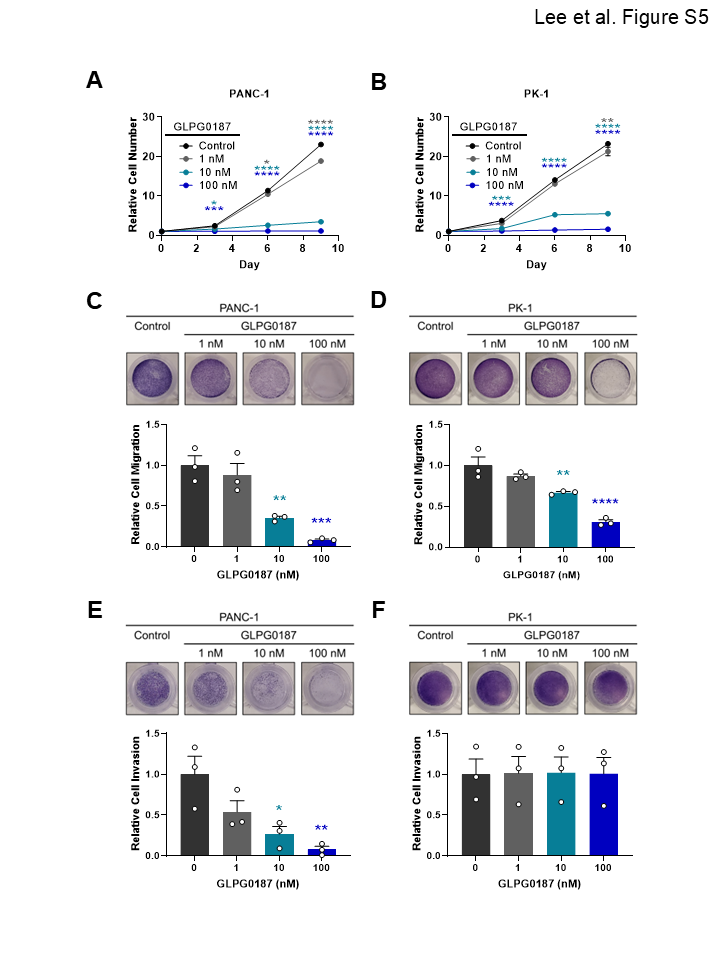

Supplement: Supplementary file 5 — Fig. S5. Related to Fig. 5. αV integrin inhibition phenotypes supporting information. Proliferation assay in (A) PANC‐1 and (B) PK‐1 cells treated with GLPG0187, showing a dose‐dependent decrease in proliferation. Each point represents the mean ± SEM from n = 3 independent experiments. *P < 0.05, **P < 0.01, ***P < 0.001, ****P < 0.0001 from Control by Two‐Way ANOVA with Dunnett's Post‐Hoc. Transwell migration assay in (C) PANC‐1 and (D) PK‐1 cells treated with GLPG0187, showing a dose‐dependent decrease in migration. Representative images of stained, migrated cells were taken 48 h after seeding. Bars represent mean ± SEM from n = 3 independent experiments. **P < 0.01, ***P < 0.001, ****P < 0.0001 from Control by One‐Way ANOVA with Dunnett's Post‐Hoc. Transwell invasion assay in (E) PANC‐1 and (F) PK‐1 cells treated with GLPG0187, showing a dose‐dependent decrease in invasion only in PK‐1 cells. Representative images of stained, invaded cells were taken 48 h after seeding. Bars represent mean ± SEM from n = 3 independent experiments. *P < 0.05, **P < 0.01 from Control by One‐Way ANOVA with Dunnett's Post‐Hoc. [file MOL2-19-3342-s003.tiff]
